# Preparation, Characterization and Catalytic Activity of Nickel Molybdate (NiMoO_4_) Nanoparticles

**DOI:** 10.3390/molecules23020273

**Published:** 2018-01-29

**Authors:** Hicham Oudghiri-Hassani, Fahd Al Wadaani

**Affiliations:** 1 Chemistry Department, College of Science, Taibah University, Almadinah 30002, Saudia Arabia; fwadaani@taibahu.edu.sa; 2 Département Sciences de la nature, Cégep de Drummondville, 960 rue Saint-Georges, Drummondville, QC J2C 6A2, Canada

**Keywords:** nickel molybdate, nanoparticles, catalysis, reduction of nitrophenol

## Abstract

Nickel molybdate (NiMoO_4_) nanoparticles were synthesized via calcination of an oxalate complex in static air at 500 °C. The oxalate complex was analyzed by thermal gravimetric analysis (TGA) and Fourier transform infrared spectroscopy (FTIR). The as-synthesized nickel molybdate was characterized by Brunauer–Emmett–Teller technique (BET), X-ray diffraction (XRD), and transmission electron microscopy (TEM) and its catalytic efficiency was tested in the reduction reaction of the three-nitrophenol isomers. The nickel molybdate displays a very high activity in the catalytic reduction of the nitro functional group to an amino. The reduction progress was controlled using Ultraviolet-Visible (UV-Vis) absorption.

## 1. Introduction

**Figure 1:**
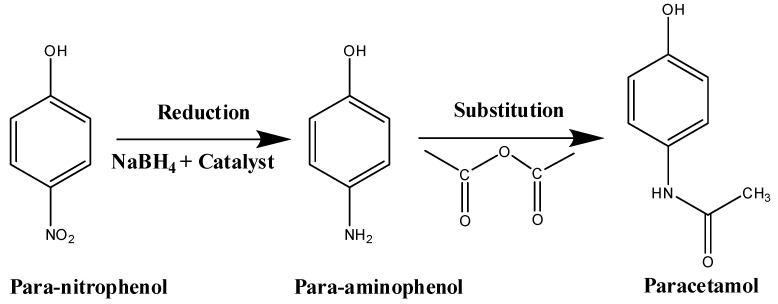
Schematic synthesis of the paracetamol from paranitrophenol.

The increasing industrial activity produces effluents containing large amounts of organic pollutants such as paranitrophenol, which was classified as priority pollutant [1]. Its reduction will decrease its toxicity. Moreover, the reduction of the paranitrophenol to paraaminophenol is an important step in the industrial production of pharmaceutical analgesics such as paracetamol or acetaminophen (Figure 1) [2].

The first step did not occur spontaneously; it requires the use of a catalyst. On the other hand, aminophenols are important pieces in the synthesis of metal-complex dyes and are also used in polymer production, hair-dying agents and in corrosion inhibition [3,4]. Due to their diverse applications, it becomes then useful and of interest to find a good and low-cost catalyst that can be used in the development of efficient processes for the synthesis of aminophenols.

Recently, nickel molybdate (NiMoO_4_) was intensively pursued because of its many applications. This promising compound was used as a catalyst for hydrodesulfurization reactions proposed by Brito et al. who studied the efficiency of the α-NiMoO_4_ and β-NiMoO_4_ in this reaction [5]. The oxidative dehydrogenation of light alkanes [6,7,8,9,10] such as propane reported by Baoyi and coworkers shown a better catalytic performance under low temperature for β-NiMoO_4_ prepared by the sol gel method compared to that the α-NiMoO_4_ prepared by the hydrothermal method [11]. Bettahar et al. tested the NiMoO_4_ as a catalyst in the partial oxidation of hydrocarbons such as propene or propylene [12]. The nickel molybdate was also studied for supercapacitor application by Yao et al. and Liu et al. [13,14,15]. NiMoO_4_ found also it applicable in the photocatalytic degradation of organic dyes such as methyl orange proposed by Alborzi et al. Mosleh et al. [16,17] or methylene blue studied by Yang et al. [18]. Nickel molybdate has attractive structures, higher specific capacitance, higher electrochemical activity and magnetic properties than other molybdates of (Zn, Co, Mg etc.) because it displays high density of states near the top of the valence band [19,20,21]. It can be found in three crystalline forms under atmospheric pressure, α-NiMoO_4_ at low temperature, β-NiMoO_4_ at high temperature, NiMoO_4_nH_2_O hydrate, and another allotrope (NiMoO_4_-II) at high pressure.

So far, several methods were reported in literature to synthesize the nickel molybdate. For example, Moreno and coworkers synthesized the β-NiMoO_4_ by using combustion process [22]. Kang and coworkers fabricated the NiMoO_4_·H_2_O nanorods with one-dimensional structures that are prepared by a facile chemical co-precipitation method [23]. Jiang et al. also synthesized the NiMoO_4_·H_2_O nanoclusters with one-dimensional nanorods via a facile and rapid microwave assisted method [24]. In the same way, a hydrothermal method was reported by Wang et al. [25] for the synthesis of hierarchical mesoporous NiMoO_4_ nanosheets, while Masteri et al. synthesized NiMoO_4_ nanocrystals via an emulsion method [26]. However, Alborzi et al. synthesized the nickel molybdate nanoparticle by sonochemical method using ammonium molybdate and nickel nitrate hexahydrate without adding surfactant [16]. Mosleh reported a facile approach to synthesize nanocrystalline NiMoO_4_ in the presence of amino acids as capping agent [17]. A sol-gel method was also used for the preparation of the NiMoO_4_ by Baoyi and coworkers [11] that shows a better catalytic activity for oxidative dehydrogenation of propane. Different shapes were reported going from nanospherical, nanorods to nanosheets [15,27,28,29]. However, all of the previously reported methods required a strict reaction conditions and high temperature or high pressure. On the other hand, the use of nickel containing catalyst is promising for several applications such as reported by Tahir et al. who suggested the NiO/Co_3_O_4_ as efficient electrocatalyst [30]. Moreover, Gentil et al. shown that a mononuclear nickel bis-diphosphine complexes exhibit reversible electrocatalytic activity for the H_2_/2H^+^ interconversion to be used in hybrid hydrogen/ air fuel cells [31].

In this study, the nickel molybdate nanoparticles were synthesized using a new method by reacting only ammonium molybdate, nickel nitrate hexahydrate and oxalic acid in the solid sate a low temperature. The new method is very simple to conduct the preparation is done without the use of any solvent. The as-prepared nickel molybdate nanoparticles were tested as catalysts in the reduction of the three nitrophenol isomers (paranitrophenol 4-NP, metanitrophenol 3-NP and orthonitrophenol 2-NP) by NaBH_4_. The results of the catalytic reaction tests are presented.

## 2. Experimental

### 2.1. Catalyst Preparation

The nickel molybdate nanocatalyst was synthetized in two steps. First, a well-ground mixture of nickel nitrate Ni(NO_3_)_2_·6H_2_O, ammonium molybdate (NH_4_)_6_Mo_7_O_24_·4H_2_O, and oxalic acid H_2_C_2_O_4_·2H_2_O in the molar ratio 1/0.143/10 [32] was used to obtain an oxalate precursor after heating at 160 °C. All chemicals were obtained from Sigma-Aldrich and used as received in the solid state.

In fact, the oxalic acid was used in excess in order to reduce molybdenum and nitrate anions and to form a coordination complex of molybdenum and nickel. The oxidation–reduction reactions and complex formation take place in the solid state when heating on a hotplate. The moisture due to water crystallization in oxalic acid and the nitrate salts play major role in all these transformations. Some preparations with anhydrous oxalic acid did not give good results. The appearance of light-green color for the nickel molybdenum complex, and the production of the NO_2_ gas (orange/brownish color) after heating at 160 °C are results of the reduction reactions of molybdenum VI and the nitrate anion NO_3_^−^ respectively. The last step was the thermal decomposition of the obtained nickel molybdenum complex for two hours under static air at 500 °C in a tubular furnace open both sides to obtain the nickel molybdate [33,34].

### 2.2. Characterization

The synthesized precursor was analyzed by thermogravimetric analysis (TGA) and differential thermal analysis (DTA) using a SDT Q 600 instrument (Ta Instruments, New Castle, NC, USA), and by Fourier transform infrared spectroscopy (FTIR) using a Shimadzu 8400S apparatus, (Shimadzu, Tokyo, Japan), at the frequency range of 400–4000 cm^−1^ using the sample that was prepared as KBr pellet. On other hand, X-ray diffractometer 6000 (Shimadzu, Tokyo, Japan), equipped with λCu-Kα = 1.5406 Ǻ with a Ni filter was used to identify the crystallized particles of the prepared nanocatalyst in the range of 10°–80° in 2θ. The Scherer equation DXRD = 0.9 λ/(B cosθ), was used to calculate the presumed spherical particle size, where θ is the Bragg angle, B is the full width at half maximum (FWHM) expressed in radians, and λ is the Cu-Kα wavelength. A Micromeritics ASAP 2020 surface area and porosity analyzer, (Micromeritics, Norcross, GA, USA), was used to measure the adsorption–desorption isotherms, and calculate the particle size with the following equation: DBET = 6000/d.S where S is the specific surface area, and d is the density.

A JEM-1400 electron microscope, (JEOL, Peabody, MA, USA), was used to reach the shape and size of the particles, while the Varian Cary 100 spectrometer, (Varian Inc., Palo Alto, CA, USA), was used to measure the evolution of the solution concentration during the reduction reaction of the three-nitrophenol isomers.

### 2.3. Test of Nitrophenol Isomers Reduction

The reduction reaction of the three-nitrophenol isomers (4-NP, 3-NP, and 2-NP) was used to test the catalytic performance of nickel molybdate. In a typical test, 40 mL of the nitrophenol isomer aqueous solution 4 × 10^−4^ M was poured into, 40 mL of sodium tetrahydroborate NaBH_4_ aqueous solution 8 × 10^−4^ M under continuous stirring at room temperature. A dark yellow color appears due to the formation of the nitrophenolate ion, and an absorption peak appears located at 401 nm, 393 nm, and 415 nm for 4-NP, 3-NP, and 2-NP respectively. The nickel molybdate nanocatalyst (0.1 g) was then added to the aqueous solution under stirring. The disappearance of the yellow color of the solution under the effect of the catalyst was followed by a UV-Vis spectrophotometer.

## 3. Results and Discussion

### 3.1. Characterizations of the Complex

**Figure 2:**
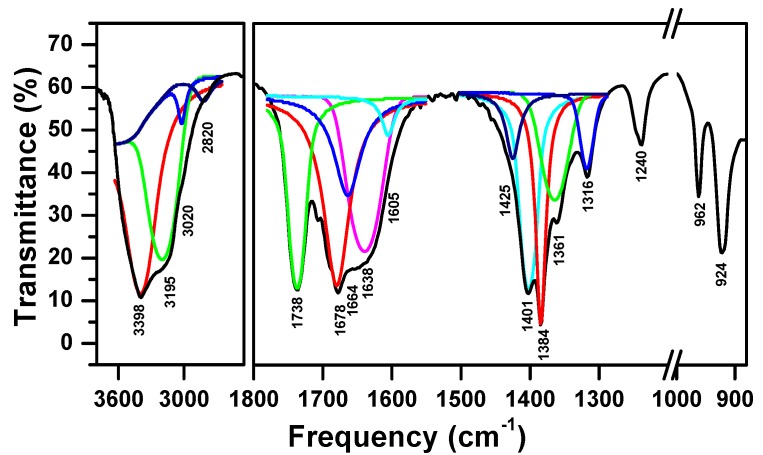
Fourier transform infrared spectrum of the synthesized complex from a mixture of nickel nitrate Ni(NO_3_)_2_·6H_2_O, ammonium molybdate (NH_4_)_6_Mo_7_O_24_·4H_2_O, and oxalic acid H_2_C_2_O_4_·2H_2_O at 160 °C.

The FTIR spectroscopy was used to identify the functional groups present in the complex synthesized by the solid-state reaction of the nickel nitrate, the ammonium molybdate and the oxalic acid, well ground mixture heated at 160 °C. In fact, the Infra-red spectrum IR given in Figure 2 shows the presence of several wide bands. The deconvolution of these bands reveal, bands at 1738 cm^−1^ and 1678 cm^−1^, which can be assigned to the C=O vibration of the oxalate group [35]. This attribution is in accordance with the existence of the C–O stretch [35] located at 1401 cm^−1^. While, both bands situated at 1361 and 1316 cm^−1^ can be assigned to υ(C–O) and δ(OCO) respectively [36].

The spectrum shows the presence of the ammonium ion and de ammonia via the presence of the symmetric and asymmetric deformation modes bands of both entities at 1664 δ_s_(NH_4_^+^), 1425 δ_as_(NH_4_^+^), and at 1605 δ_s_(NH_3_), 1240 cm^−1^ δ_as_(NH_3_) respectively. In the NH stretching region, the spectrum shows the presence of the bands at 3195 cm^−1^ and at 3020 and 2820 cm^−1^ that can be assigned to coordinated ammonia and to ammonium ions, respectively. These attributions were in accordance with the study of Ramis and coworkers [37] and Wen and coworkers [38]. At high frequencies, the FTIR spectrum shows also a band at 3398 cm^−1^ that corresponds to O–H bridging group between two metal ions [39,40]. On the other hand, the spectrum shows an absorbance bands at 1384 cm^−1^, which is assigned to the δ(OH) [40], while that situated at 1638 cm^−1^ was attributed to δ(H_2_O) [41]. Moreover, the spectrum shows also, the Mo=O stretch [35] via the presence of the bands at 924 cm^−1^, and 962 cm^−1^. These results confirm the existence of the functional groups of oxalate, hydroxyl (–OH), water, oxo (Mo=O), NH_3_, and NH_4_^+^ ion in the synthesized complex.

**Figure 3:**
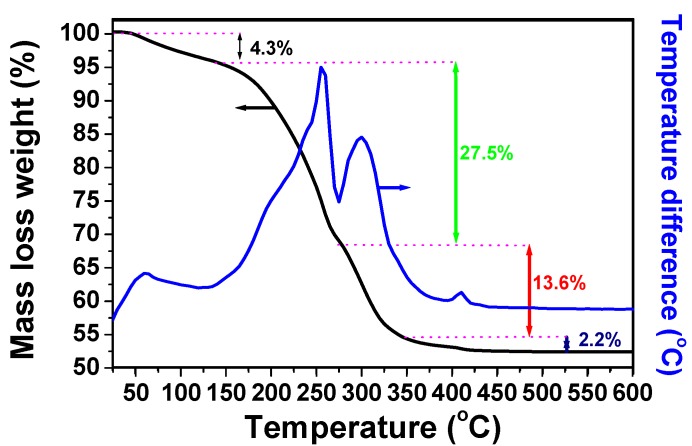
Thermal gravimetric and thermal differential curves of the synthesized complex from a mixture of nickel nitrate Ni(NO_3_)_2_·6H_2_O, ammonium molybdate (NH_4_)_6_Mo_7_O_24_·4H_2_O, and oxalic acid H_2_C_2_O_4_·2H_2_O at 160 °C.

The thermogravimetric analysis was performed on the obtained complex in static air (Figure 3). The recorded curve can be divided in four parts. In the first part, a 4.3% weight loss was observed until 150 °C, which can be due to water molecules existing in the complex, confirmed by infrared spectroscopy studies reported above. In the second and third parts, two strongly and rapid exothermic losses occurs between 150 and 350 °C corresponding to the decomposition of the complex and to a weight loss of 41.1%. In the fourth and last part, the curve shows a small and final loss between 350 and 450 °C with a mass loss of 2.2%. A similar loss in the same range was also obtained in the previous study of bismuth oxalate complex that can be attributed to OH group [39]. By compiling the results obtained by FTIR, TGA and the possible oxidation degree of nickel and molybdenum, a formula of the oxalate complex can be suggested as (NH_3_)(NH_4_)NiMoO(C_2_O_4_)_2_(OH)·H_2_O. The total weight loss observed is of 47.6% in comparison with the theoretical value of 47.5% for the suggested formula. The temperature of 500 °C was chosen to obtain the nickel molybdate by the calcination of the complex in static air.

### 3.2. Nickel Molybdate Characterization

#### 3.2.1. X-ray Diffraction

**Figure 4:**
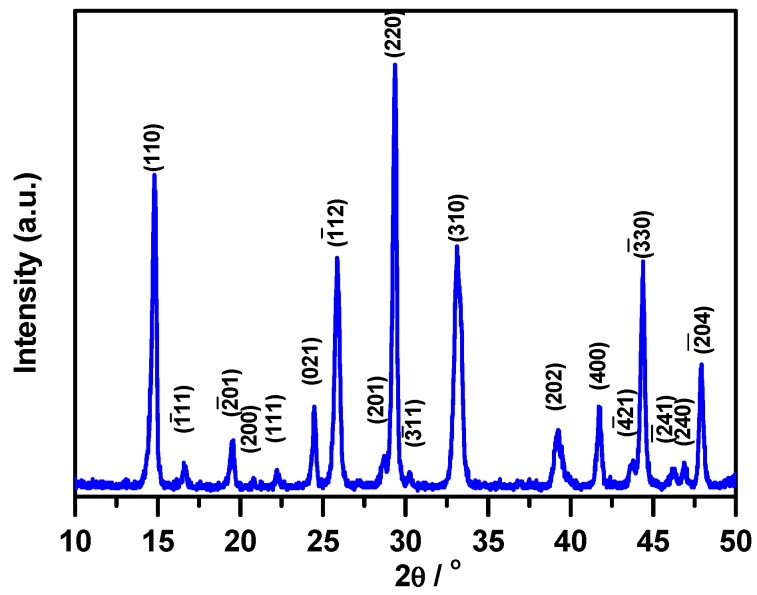
X-ray diffraction pattern of the synthesized nickel molybdate, NiMoO_4_, obtained after calcination of the oxalate complex at 500 °C.

The powder obtained after the calcination of the complex at 500 °C was analyzed by the X-ray diffraction technique (XRD) and the recorded pattern is presented in Figure 4. The XRD pattern is indexed in accordance with JCPDS file # 31-0902, which corresponds to the monoclinic phase α-NiMoO_4_ that crystallizes in the space group C2/m (12) with the parameters a = 9.592 Ǻ, b = 8.755 Ǻ, and c = 7.655 Ǻ and β = 114.24°.

The intense peak located at 2ϴ = 14.8° (110) which corresponds to the highest d spacing was chosen to calculate the crystallites size D_XRD_, that was found to be of 18 nm.

#### 3.2.2. Specific Surface Area Determination

The specific surface area of the nickel molybdate NiMoO_4_ synthetized by this simple method was estimated by the Brunauer–Emmett–Teller technique (BET) [42]. It was found to be of S_BET_ = 29.86 m^2^/g. Knowing the value of the nickel molybdate density, d = 3.3723 g/cm^3^, the particle size D_BET_ was calculated to be approximately 60 nm. Calculations using BJH (Barrett, Joyner and Halenda) method permit to find a pore volume of 0.114 cm^3^/g with a pore size of 128 Å, indicating that the material has a mesoporous character [43].

#### 3.2.3. Transmission Electron Microscopy

**Figure 5:**
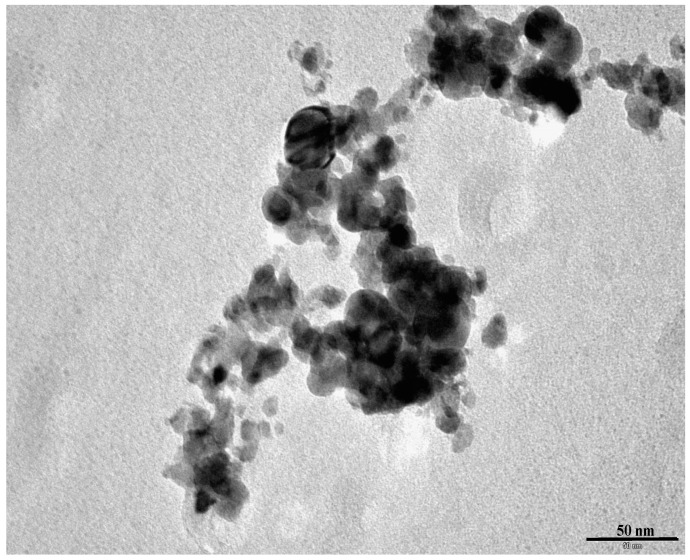
Transmission electron microscopy micrograph of the synthesized nickel molybdate, NiMoO_4_, obtained after calcination of the oxalate complex at 500 °C.

The micrograph of the nickel molybdate prepared is shown in Figure 5. The particles are spherical and of 10 to 20 nm in size. However agglomerates of these nanoparticles of about 100 nm are formed.

The calculations carried out with the XRD method on the first peak (110) show that the average size of the crystals is of 18 nm, this was consistent with the Transmission electron microscopy (TEM) observation where the particles size observed was found between 10 and 20 nm with a nonhomogeneous distribution in size. On the other hand, the high value of particles size of 60 nm, calculated using the formula D_BET_ = 6000/d.S where S is the specific surface area and d is the density, was not consistent with the obtained values from XRD and TEM. This can be explained by the fact that when the particles are agglomerated, the specific surface area tends to decrease as the allowed surface for reactivity is lowered by contact between particles. As such, the particles’ size value will increase as given by the above formula.

### 3.3. Reduction Test of Nitrophenol Isomers

**Figure 6:**
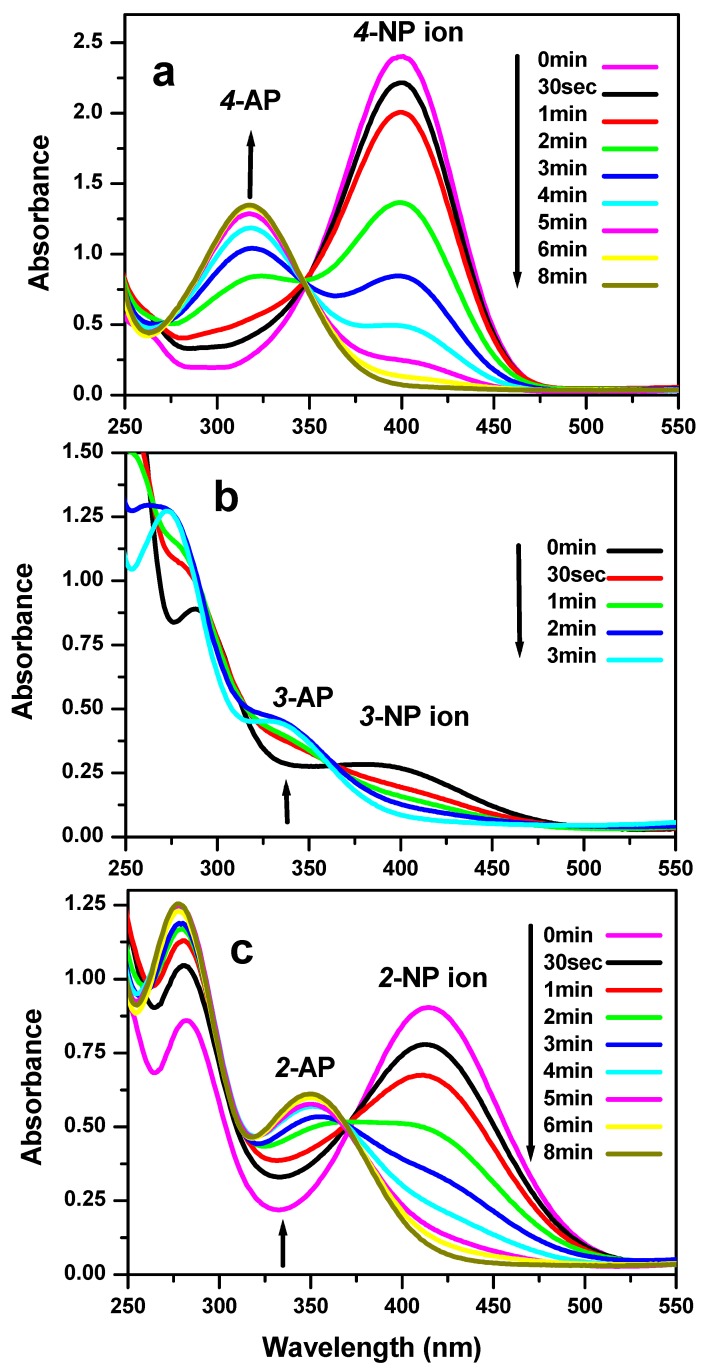
UV-visible spectra of the reduction reaction solution of (**a**) 4-nitrophenol; (**b**) 3-nitrophenol; and (**c**) 2-nitrophenol in the presence of NaBH_4_ at room temperature after adding nickel molybdate, NiMoO_4_, obtained after calcination of the oxalate complex at 500 °C.

**Figure 7:**
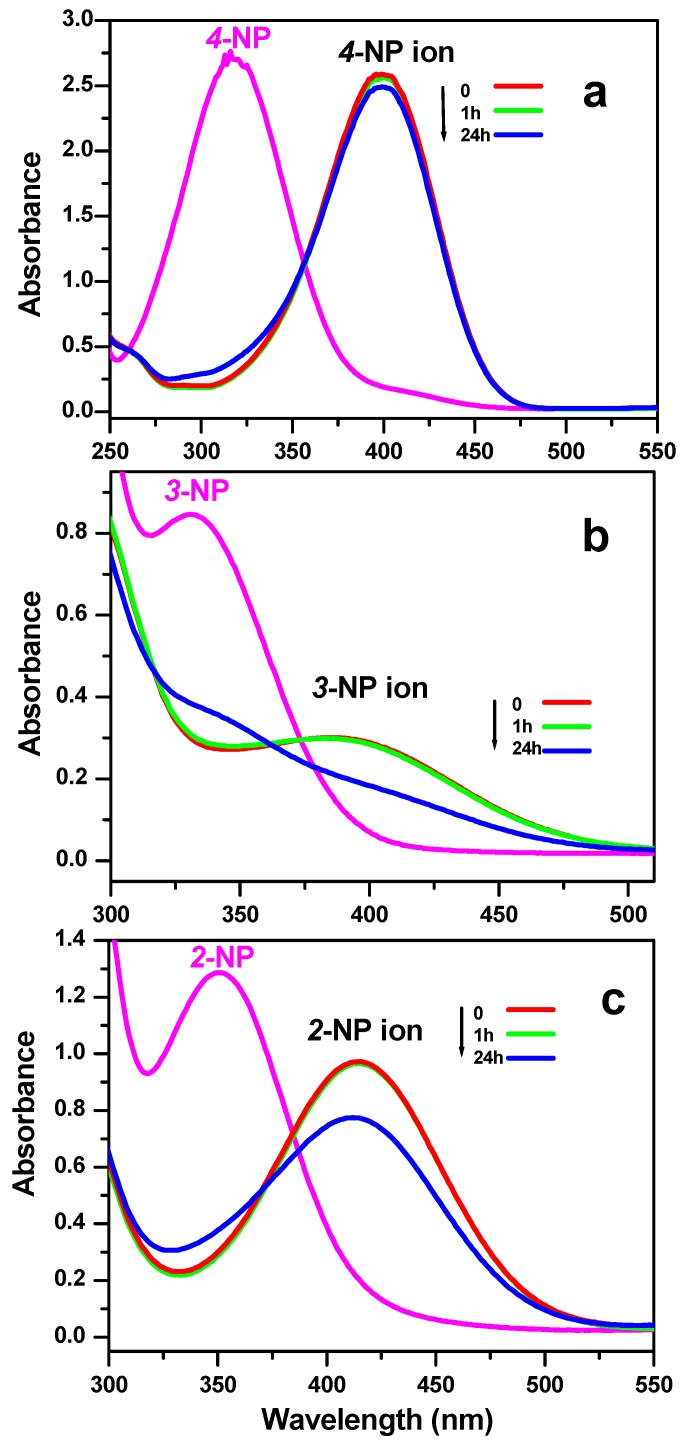
UV–vis spectra of: (**a**) 4-nitrophenol; (**b**) 3-nitrophenol; and (**c**) 2-nitrophenol (NP) isomers before and after adding NaBH_4_ without adding nanoparticles at room temperature.

The reduction reaction of three nitrophenol isomers with NaBH_4_ was investigated to test the catalytic efficiency of the successfully synthesized nickel molybdate (Figure 6a–c). Once the NaBH_4_ was added, the nitrophenol isomers were converted to the NP ion nitrophenolate isomers (Figure 7). Before the addition of the as prepared catalyst, the dark yellow color of the solution stays unchanged during a period of 24 h. However, after the addition of the nanocatalyst, the solution becomes uncolored in few minutes for all of the three-nitrophenol isomers. The higher peaks of absorption located at 401 nm, 393 nm, and 415 nm disappear in favor to new peaks situated at 317 nm, 328 nm, and 347 nm for the 4-NP, 3-NP and 2-NP, respectively. In fact, 8 min, 3 min, and 8 min were the necessary time to achieve the reaction with the appearance of the corresponding aminophenol isomers at room temperature. This result demonstrates the high catalytic efficiency of the synthesized nickel molybdate in the reduction of the nitrophenol isomers compared to previous research works found in the literature as presented in Table 1.

**Figure 8:**
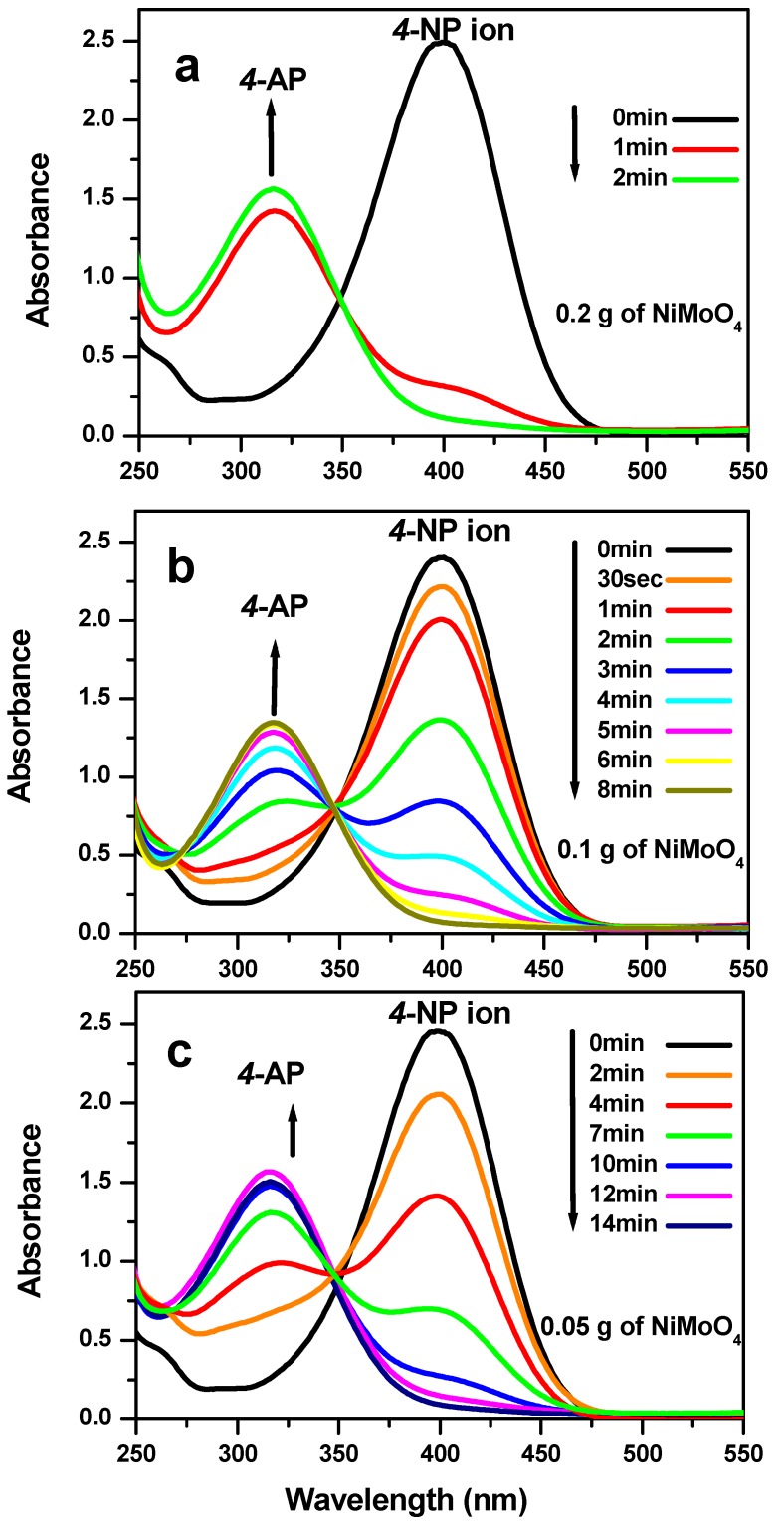
UV–vis spectra of reduction reaction for 4-nitrophenol using different amount of nickel molybdate, NiMoO_4_, (**a**) 0.20 g; (**b**) 0.10 g; and (**c**) 0.05 g at room temperature.

In order to test the influence of the nanoparticles ratio on the reduction reaction rate an experiment was conducted on the reduction of the 4-NP isomer taken as example. Three different amounts of 0.05 g, 0.1 g and 0.2 g were used in the protocol cited above. The results are presented in Figure 8. The time to achieve the reduction reaction was found to be of 14 min, 8 min and 2 min respectively. It shown that the reaction is faster when the amount of catalyst is increased.

A mechanism for this reduction reaction can be supposed as follows. The nickel molybdate nanoparticles (NiMoO_4_) dissociated the BH_4_^−^ to form (NiMoO_4_)-H and (NiMoO_4_)-BH_3_^−^ as reactive intermediates (Equation (1)) [47,48]. Afterward, these intermediates reduce the nitrophenol isomers by (Equations (2) and (3)). Six electrons are involved in the formation of the aminophenol isomers (APi) from the corresponding nitrophenol (NPi).

2NiMoO_4_ + BH_4_^−^ → (NiMoO_4_)-H + (NiMoO_4_)-BH_3_^−^ (1)

6(NiMoO_4_)-H + NPi → APi + 6NiMoO_4_ + 6H^+^ (2)

6(NiMoO_4_)-BH_3_^−^ + NPi → APi + 6NiMoO_4_ + 6BH_3_ (3)

## 4. Conclusions

The nickel molybdate, α-NiMoO_4_, was satisfyingly prepared as nanoparticles using a new and simple method. The high efficiency of the as-prepared nanocatalyst was confirmed in the reduction of the 4-NP, 3-NP, and 2-NP nitrophenol isomers. The studied nickel molybdate can be presented as a potential catalyst candidate for the reduction of the nitro functional group to an amino group.

## Figures and Tables

**Table 1:** A comparison of reaction time for the reduction of 2-NP 3-NP and 4-Np by NiMoO_4_ with other nanocatalysts reported in the literature.

Catalyst	Type	Concentration of NP (mol/L)	Reaction Time (min)	References
NiMoO_4_	Nanoparticles	2 × 10^−4^	8 for 4-NP 3 for 3-NP 8 for 2-NP	This work
CuFe_2_O_4_	Nanoparticles	3.6 × 10^−5^	4 for 4-NP 5 for 3-NP 3 for 2-NP	[44]
NiFe_2_O_4_	Nanoparticles	3.6 × 10^−5^	38 for 4-NP 36 for 3-NP 28 for 2-NP	[44]
CuO/γAl_2_O_3_	Nanocomposites	2.9 × 10^−5^	12 for 4-NP 20 for 3-NP 15 for 2-NP	[45]
Ni/C black	Nanocomposites	5.0 × 10^−4^	15 for 4-NP 15 for 3-NP 15 for 2-NP	[46]
